# Engineered human platelet-derived microparticles as natural vectors for targeted drug delivery

**DOI:** 10.18632/oncotarget.27223

**Published:** 2019-10-08

**Authors:** Jyotsna Kailashiya, Vineeta Gupta, Debabrata Dash

**Affiliations:** ^1^ Department of Biochemistry, Institute of Medical Sciences, Banaras Hindu University, Varanasi, Uttar Pradesh 221005, India; ^2^ Department of Pediatrics, Institute of Medical Sciences, Banaras Hindu University, Varanasi, Uttar Pradesh 221005, India

**Keywords:** cancer targeting, drug-delivery vector, doxorubicin, leukemia, platelet-derived microparticles

## Abstract

Drug targeting has opened a new paradigm in therapeutics with development of delivery vectors like liposomes and polymeric nanoparticles. Although their clinical application is crippled by limited biological adaptability. Off-target toxicity and biocompatibility still remains one of the critical problems in anticancer therapeutics that can be life-threatening. Here we report a quick, simple and facile method of engineering human platelets to generate drug loaded platelet-derived microparticles (PMPs) by top-down approach, which are biocompatible and naturally target leukemia cells. Drug loaded PMPs and cancer cell uptake were characterized by flow cytometry, confocal microscopy, Nanoparticle Tracking Analysis and fluorimetry. Effective drug delivery was tested in cancer cell lines as well as in clinical samples from leukemia patients. We explored that PMPs are capable of carrying multiple drug payloads, have long shelf life and can be harvested in large quantity in short period. Importantly, PMPs exhibited remarkably higher toxicity towards cancer cells than free drug and had lower escape into extravascular spaces. Transfer of drug to cancer cells of leukemia patients was significantly higher than free drug, when delivered through PMPs. Our experiments validated therapeutic application of PMPs as biocompatible drug delivery vector against cancer cells with minimal off-target delivery.

## INTRODUCTION

Off-target toxicity is presently one of the main issues associated with drug administration, especially anticancer therapy [1]. Being part of health care delivery system, we often encounter adverse effects of chemotherapeutic agents due to interaction with off-target tissues. Frequently, these unwanted outcomes become more serious health problems than disease itself. For example, anticancer therapy can trigger severe anemia due to bone marrow suppression, cachexia, nerve damages causing visual and hearing losses and organ failures. This indicates large lacunae in effective targeted drug delivery techniques.

Targeting drugs to specific sites of action through nano-sized vehicles has opened new paradigm in therapeutics [2]. Despite development of synthetic drug delivery vectors such as liposomes and polymeric nanoparticles; their comprehensive adaptability to biological environment, metabolism and clearance from body remains uncontrolled [2, 3]. Apart from these man-made approaches, it was recently highlighted that nature possesses its own internal cargo delivery system [4, 5], which deploys extracellular microvesicles (including microparticles and exosomes) as conveyors of cellular materials [6, 7]. Microvesicles participate in cell to cell communication and transfer of information [5].

Microparticles are submicron fragments released mostly from cells of circulatory system (platelets, endothelial cells, leukocytes, erythrocytes and macrophages) into blood that fuse with target cells acting as ‘natural’ biological vectors [8]. Notably, microparticles deliver payloads of lipids, proteins, miRNAs [8–10], mRNAs [11] and non-coding RNAs into recipient cells, thus modulating gene expression and function of latter. Recent studies have shown promising results in employing microparticles as novel therapeutic vehicles for delivery of RNA-interference and drugs [12–14]. Unlike its artificial synthetic counterpart liposomes, microparticles are poorly immunogenic while still capable of shielding ‘therapeutic cargoes’ from rapid degradation *in vivo*, as well as overcoming biological barriers such as the blood-brain barrier [15–17].

Activated platelets release platelet-derived microparticles (PMPs) under various physiological and pathological conditions [9, 10, 18], which circulate in blood in abundance and naturally interact with leukocytes [18–21]. In this study, we have engineered platelets to release drug-loaded PMPs by top-down procedure, that are targeted at circulating leukemia, (blood cancer) cells and demonstrate minimization of off-target toxicity with this strategy.

## RESULTS

### PMPDox characterization

**Figure 1:**
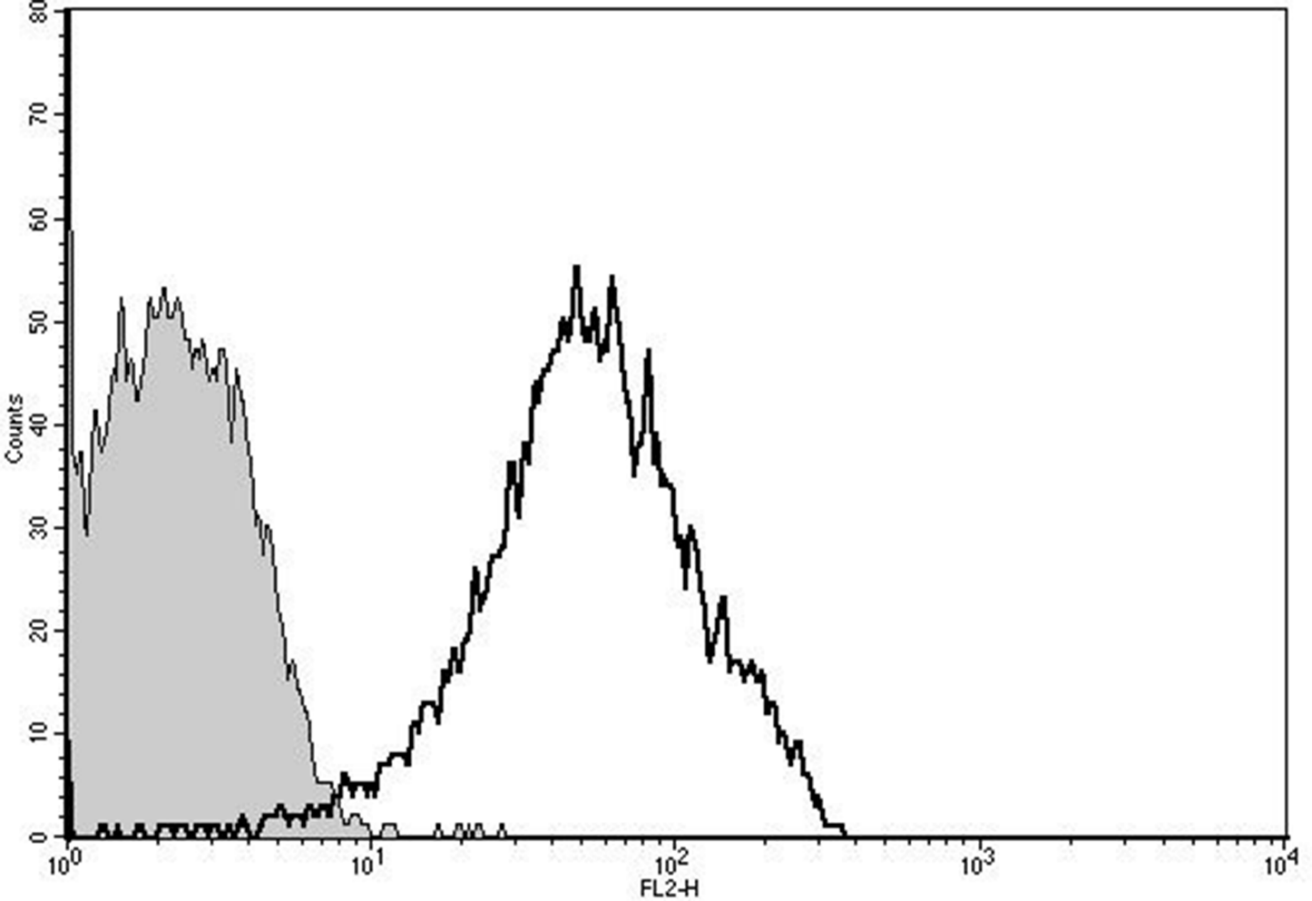
Doxorubicin loading in PMP analyzed by flow cytometry. Unshaded, PMPs carrying Dox; shaded, control PMPs without Dox. The figure is representative of 3 independent experiments.

**Figure 2:**
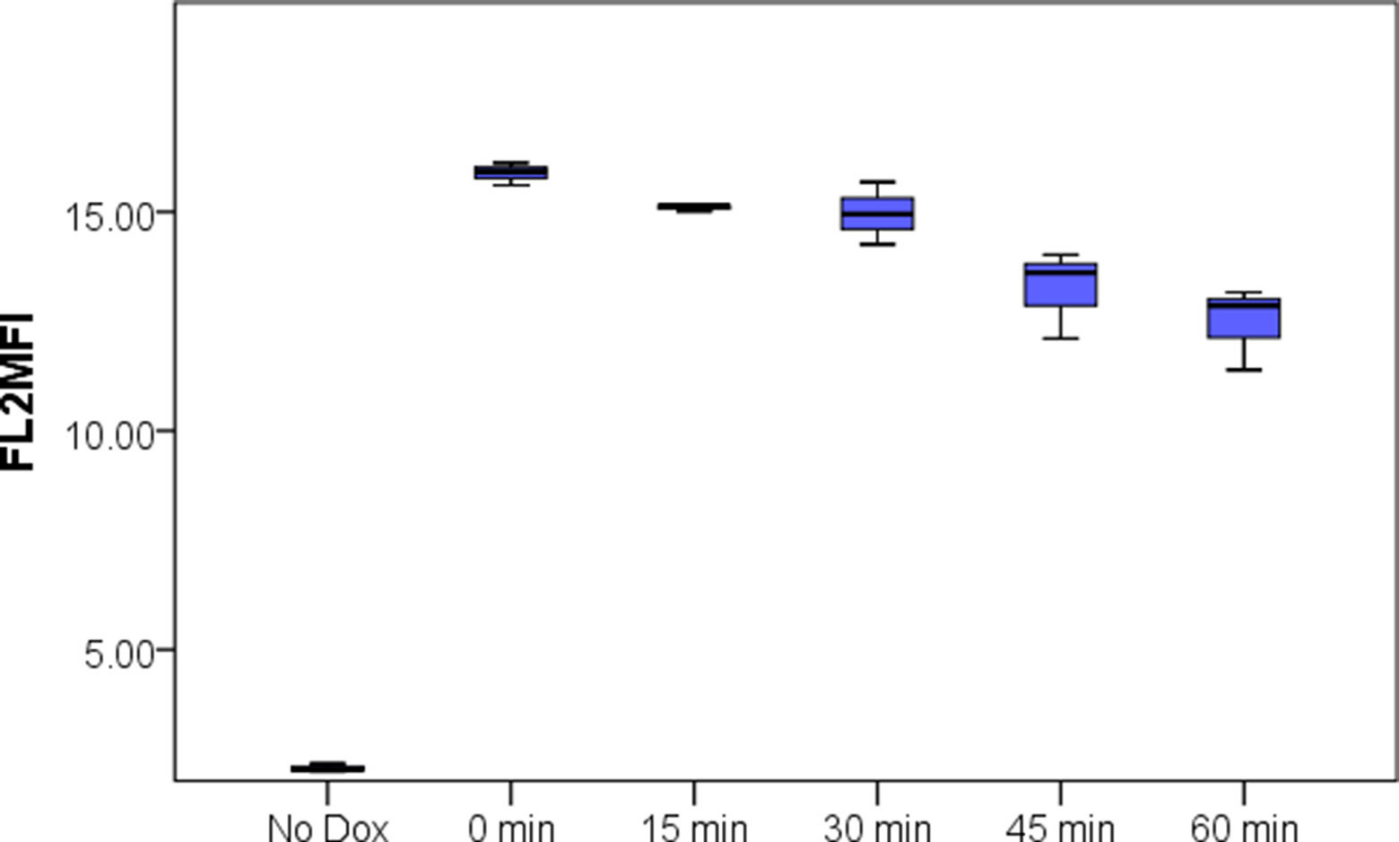
Leaching of Doxorubicin from PMPDox over 60 min period studied by flow cytometry. Box plots exhibit median, range, 1st and 3rd quartile values from triplicate experiments. The figure is representative of 3 independent experiments.

From nanoparticle tracking analysis (NTA), PMP count in prepared PMPDox (Doxorbicin loaded PMPs) samples was found to be 2.3 ± 0.5 × 10^9^ per ml. Microparticle sizes were found to range from 10-810 nm, majority of population being within 129–145 nm. Successful loading of Doxorubicin (Dox) in PMPs was confirmed from higher particle fluorescence (Figure 1). In order to evaluate amount of Dox captured, PMPDox membrane was lysed with 1% Triton X-100 and extent of Dox released was analyzed from fluorescence at 485/530 nm (ex./em.) against known drug concentrations. Dox loaded in PMPDox was found to be 2.3 ± 0.7 μg/ml in our preparation. No change in Dox content was observed upon storage of PMPDox at −80° C for up to 34 days (Supplementary Figure 1). In order to test retention / leaching of loaded Dox, we diluted PMPDox with sheath fluid (1:4, v/v) and monitored drop in FL2 signal at different time points by flow cytometry. There was a slow decay in particle fluorescence (mean fluorescence intensity or MFI) from 15.8 ± 0.25 to 12.12 ± 1.03 (*n* =3) after 1 h of dilution consistent with ~76.7% Dox retention (Figure 2).

To explore whether drugs/compounds other than Dox can be loaded into PMPs by our top-down approach, we substituted Dox with either methylene blue (1 mg/ml) or δ-aminolevulinic acid (ALA) (40 μM), which are fluorescent compounds and easily traceable in PMPs. Flow cytometry analysis demonstrated successful incorporation of both compounds in PMPs (Supplementary Figure 2), validating PMPs as efficient drug carriers.

### PMPDox-mediated delivery of doxorubicin and uptake by human leukemia cell lines

**Figure 3:**
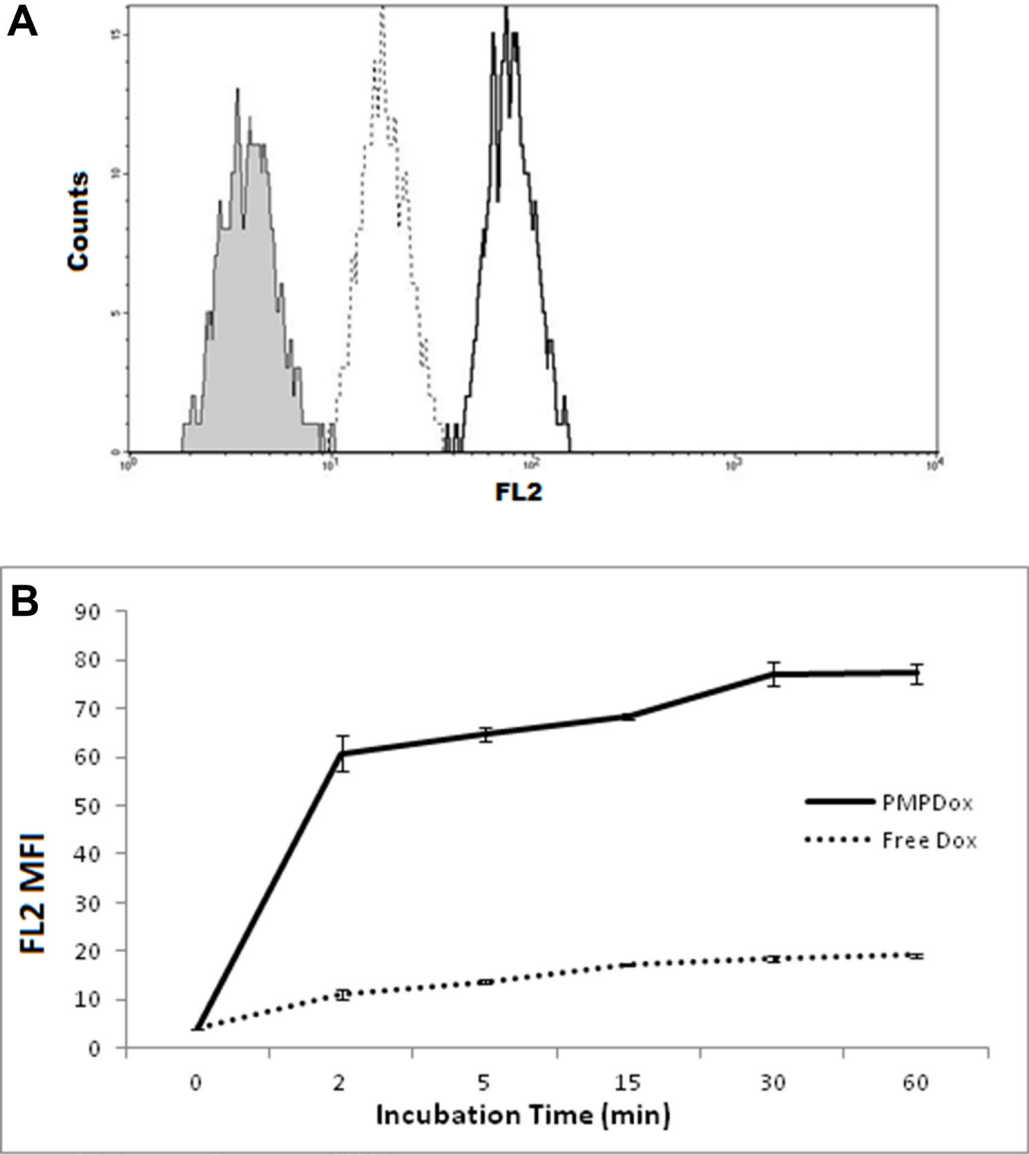
(**A**) uptake of doxorubicin by HL 60 cells following 60 min incubation with equivalent doses of either PMPDox (solid margin, unshaded) or free Dox (dotted margin, unshaded) studied by flow cytometry. Shaded curve represents HL 60 cells before exposure to drug. The figure is representative of 3 independent experiments. (**B**) Dox uptake by HL 60 cells at different time points. Graph represents mean ± SD from 3 independent experiments.

In order to evaluate PMPDox-mediated Dox delivery to leukemia cells, we incubated HL 60 cells with either free Dox (0.6 μg/ml) or PMPDox carrying equivalent drug amount (0.6 μg Dox/ml of PMPDox). Dox uptake by cells was validated from appearance of bright fluorescence localized to cell nuclei under fluorescence microscope (Supplementary Figure 3). Nearly 7 times higher amount of drug was found to be assimilated by cells exposed to PMPDox for 60 min than those incubated with free Dox (Figure 3A). At different time points incorporation of Dox in HL 60 was remarkably greater (by about 6 times after 2 min incubation) in presence of PMPDox than free Dox (Figure 3B), which could be attributable to targeted delivery of drug by PMP.

**Figure 4:**
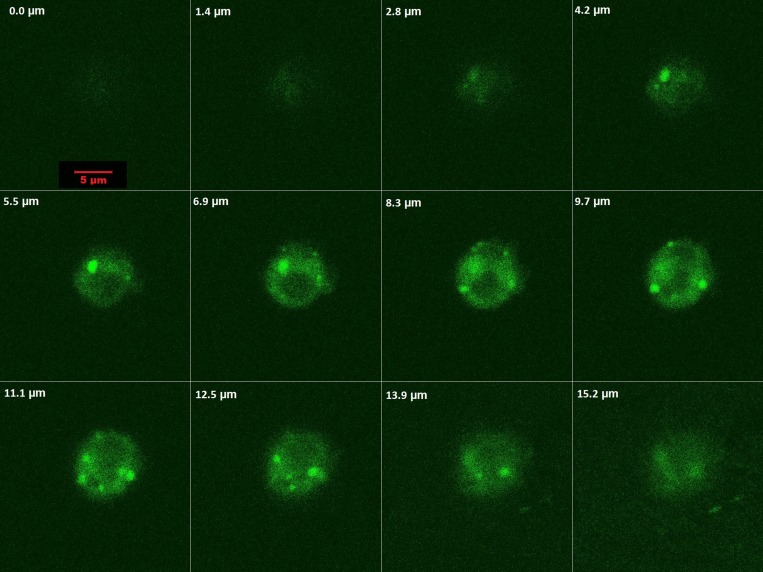
Optical slicing / Z-stacking by confocal microscopy showing intracellular localization and distribution of of PMPCalcein in HL 60 cells.

In order to characterize interaction of PMPs with leukemia cells, HL60 cells were incubated with PMPCalcein (to prevent leakage mediated fluorescence uptake, as Calcein becomes impermeable through cell membranes and only PMPs mediated uptake will be visible) for 30 min and cellular acquisition of Calcein fluorescence was evaluated by optical slicing (1.4 μm steps) / Z-stacking employing confocal microscopy. As observed in Figure 4, cytosol became diffusely fluorescent with presence of intact microparticles (having bright green fluorescence) visible within cell. Fluorescence density was maximum within the cytosol in optical sections between 5.5 and 13.9 μm. Optical slicing images (Figure 4) and 3D construct video (Supplementary Video 1) were strongly suggestive of uptake of PMPs by HL60 cells.

**Figure 5:**
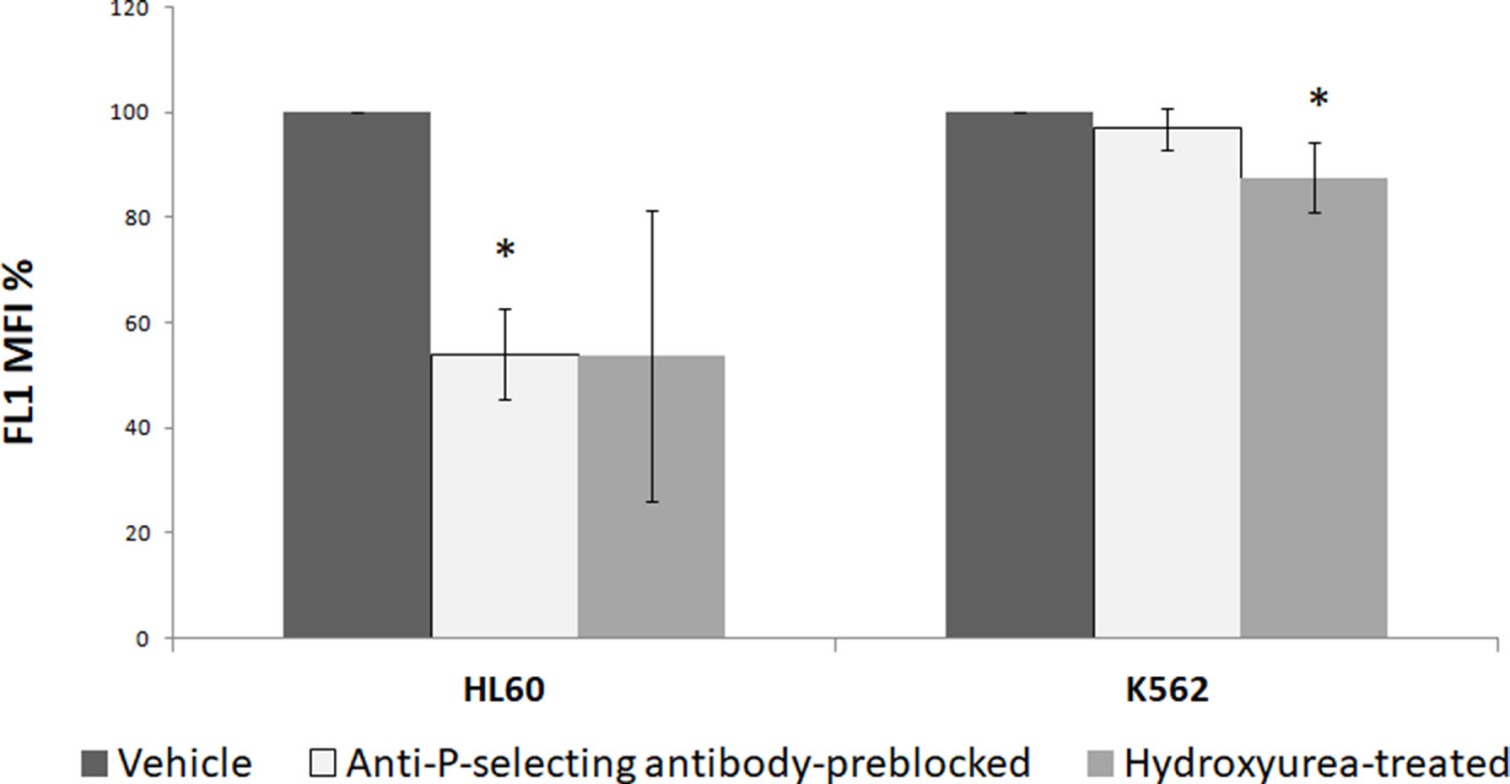
PMPs uptake by leukemia cell lines (HL 60 and K562) in presence of inhibitors of PSGL1-P-selectin interaction studied by flow cytometry. ^*^ *P* < 0.05.

PSGL1 present on surface of neutrophils and leukemia cells is the critical receptor responsible for interaction with P-selectin-bearing cells including platelets [20, 22]. In order to implicate PSGL1-P-selectin interaction in PMPs internalization by leukemic cells, HL 60 and K562 cells (100 μl each from 1 × 10^6^/ml cell suspensions) were separately incubated with 100 μl PMPCalcein for 1 h. In different experiments PSGL1-P-selectin interaction was blocked by pre-incubation of leukemia cells for 30 min with hydroxyurea (1.4 mM) prior to addition of PMPCalcein, or by incubation of PMPCalcein with anti-P-selectin antibody (5 μg in 100 μl) for 30 min before mixing with leukemia cells [20, 23]. As shown in Figure 5, uptake of PMPCalcein by HL 60 cells was significantly inhibited (by ~50%, *p* = 0.01) in presence of anti-P-selectin antibody, as well as by hydroxyurea (by ~50%, *p* = 0.05), indicative of P-selectin-PSGL1-mediated uptake of PMP by HL 60. In keeping with this, uptake of PMP by K562 (erythroleukemia) cells, which are known to have limited interaction with P-selectin [24, 25], was minimally affected by the blocking antibody or hydroxyurea. This suggests existence of multiple interaction mechanisms apart from P-selectin-PSGL interface that facilitate PMP uptake by leukemia cells.

### PMPDox mediated toxicity in human leukemia cell lines

**Figure 6:**
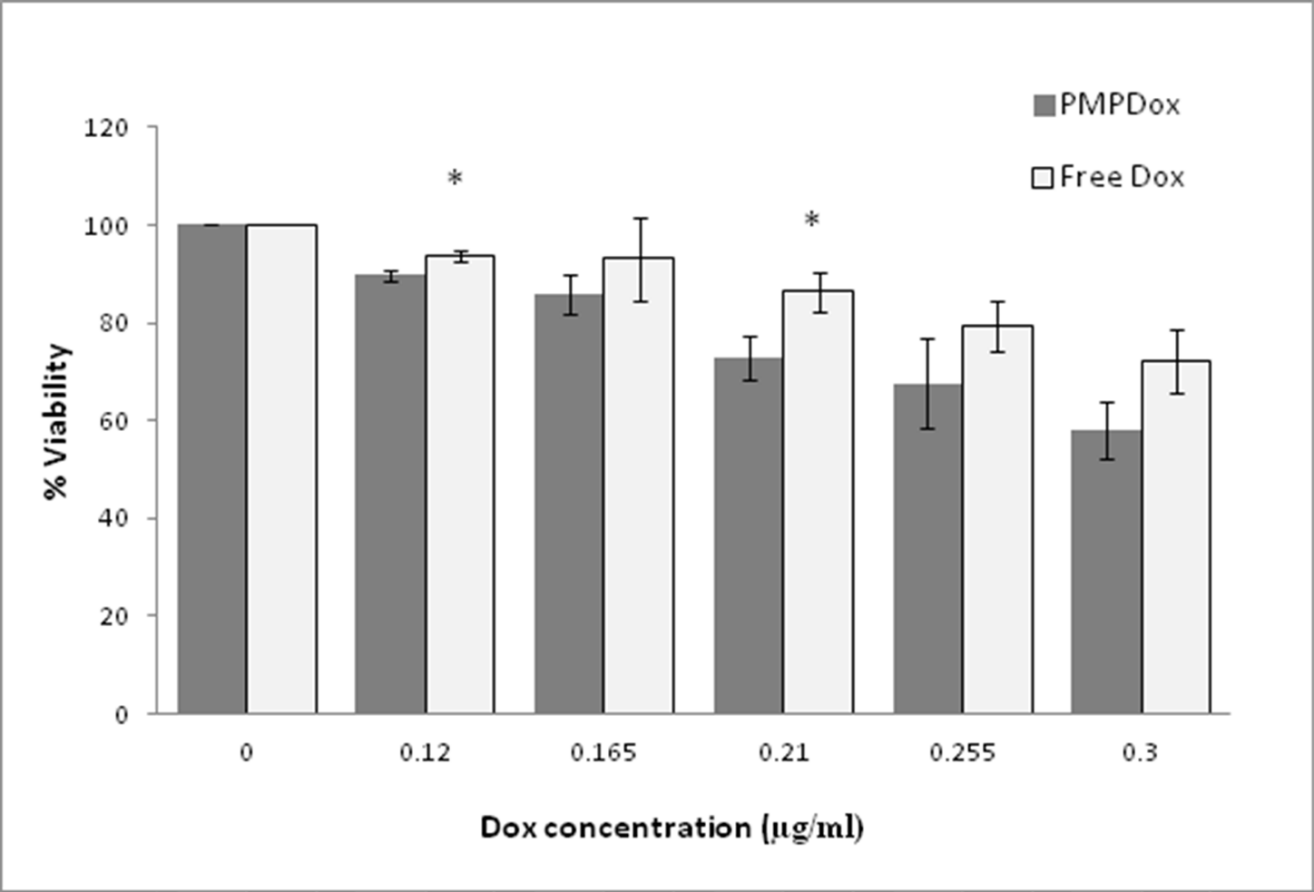
Toxicity of Doxorubicin loaded PMPs (PMPDox) and free Doxorubicin on HL 60 cells studied by MTT assay. Each bar represents mean ± SD from 3 independent experiments. ^*^ *p* < 0.05 vs PMPDox.

We next asked whether drug delivery by PMPDox would result in greater toxicity at target tissue compared to free Dox. Varying concentrations of free drug or equivalent PMPDox were incubated with HL 60 in 96-well plates for 48 h at 37° C under 5% CO_2_ and MTT assay was performed to evaluate viability of cells. As demonstrated in Figure 6, PMPDox (at Dox concentrations 0.12 and 0.21 μg/ml) was found to be remarkably more toxic to cancer cells than equivalent doses of free Dox (*p* <0.05). PMPDox also adversely affected viability of another leukemia cells line, K562, in dose-dependent manner (Supplementary Figure 4). EC50 (half maximal effective concentration) computed from interaction of drug with HL 60 cells was found to be lower (0.38 μg/ml) in case of PMPDox than that for free Dox (0.58 μg/ml), suggestive of lesser drug requirement when delivered through PMPDox.

### PMPDox and free Dox escape through endothelial cells

**Figure 7:**
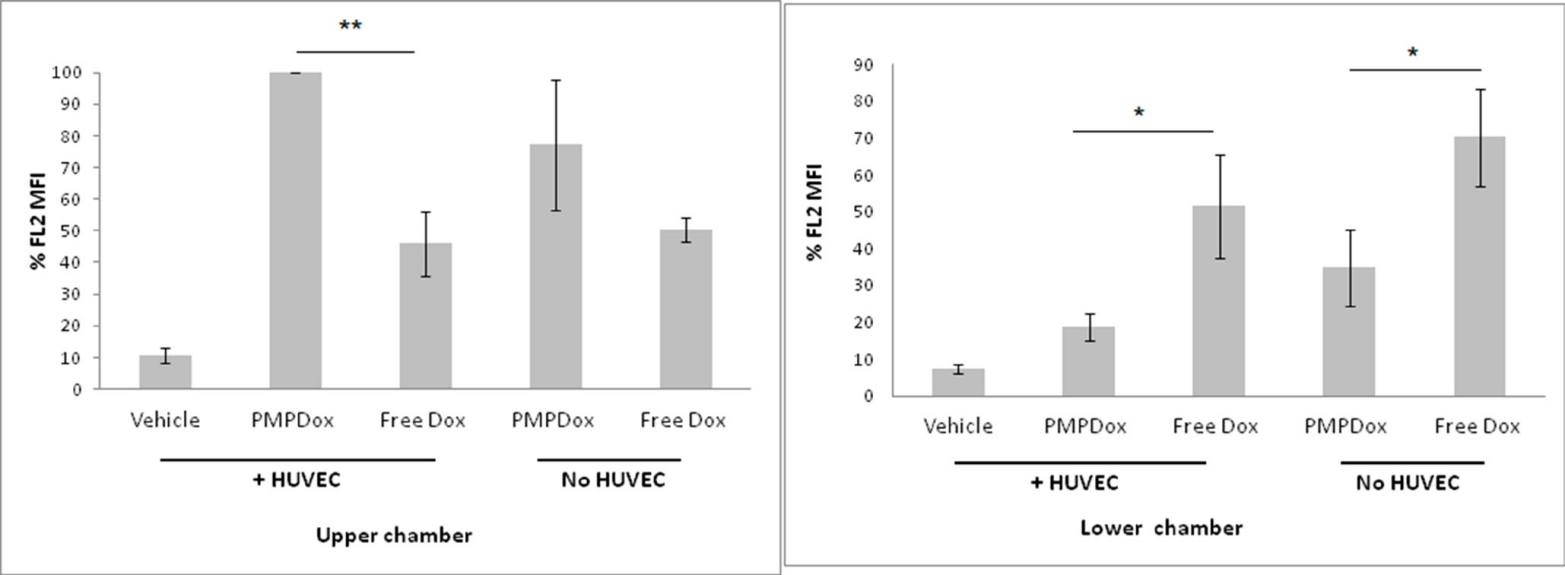
Uptake of Doxorubicin by HL 60 cells in upper and lower chambers of Transwell plates. Figures represent results from three independent experiments. ^*^ *P* < 0.05; ^**^ *P* < 0.001.

As loading of Dox in PMP enhanced targeted drug delivery, we next asked whether PMPDox reduced off-target toxicity by limiting drug escape into extravascular tissues. We employed Transwell plate system to study extravascular drug escape where two chambers in the well were separated by porous membrane (1 μm pore size) lined with or without HUVEC cells that mimicked the endothelial lining separating intravascular (upper chamber) from extravascular (lower chamber) compartments (Supplementary Figure 5). Drug delivery to HL 60 cells in both the chambers were evaluated 12 h after addition of either PMPDox or free Dox to upper chamber. As expected, uptake of Dox by HL 60 cells in upper chamber was greater when incubated with PMPDox than with equivalent amount free Dox. Interestingly, diffusion across membrane lined with HUVEC and uptake by HL 60 in lower chamber (representing extravascular space) was significantly less with PMPDox (18.8 ± 3.73%) than with free drug (51.5 ± 13.87%) (Figure 7), suggestive of restricted extravascular mobilization of drug when delivered by PMPDox. Even in absence of HUVEC lining (representing sites of endothelial damage) diffusion to lower chamber was lower with PMPDox compared to free Dox (Figure 7). Thus, drug delivery to leukemia cells by PMPDox-based approach is much safer, as possibility of extravasation of free drug and off-target tissue toxicity is reduced. In keeping with these observations, exosome-based drug delivery has already been shown to be associated with reduced cardiac and hepato-toxicity in animal models [13].

### Doxorubicin uptake by leukemia cells in human clinical samples

**Figure 8:**
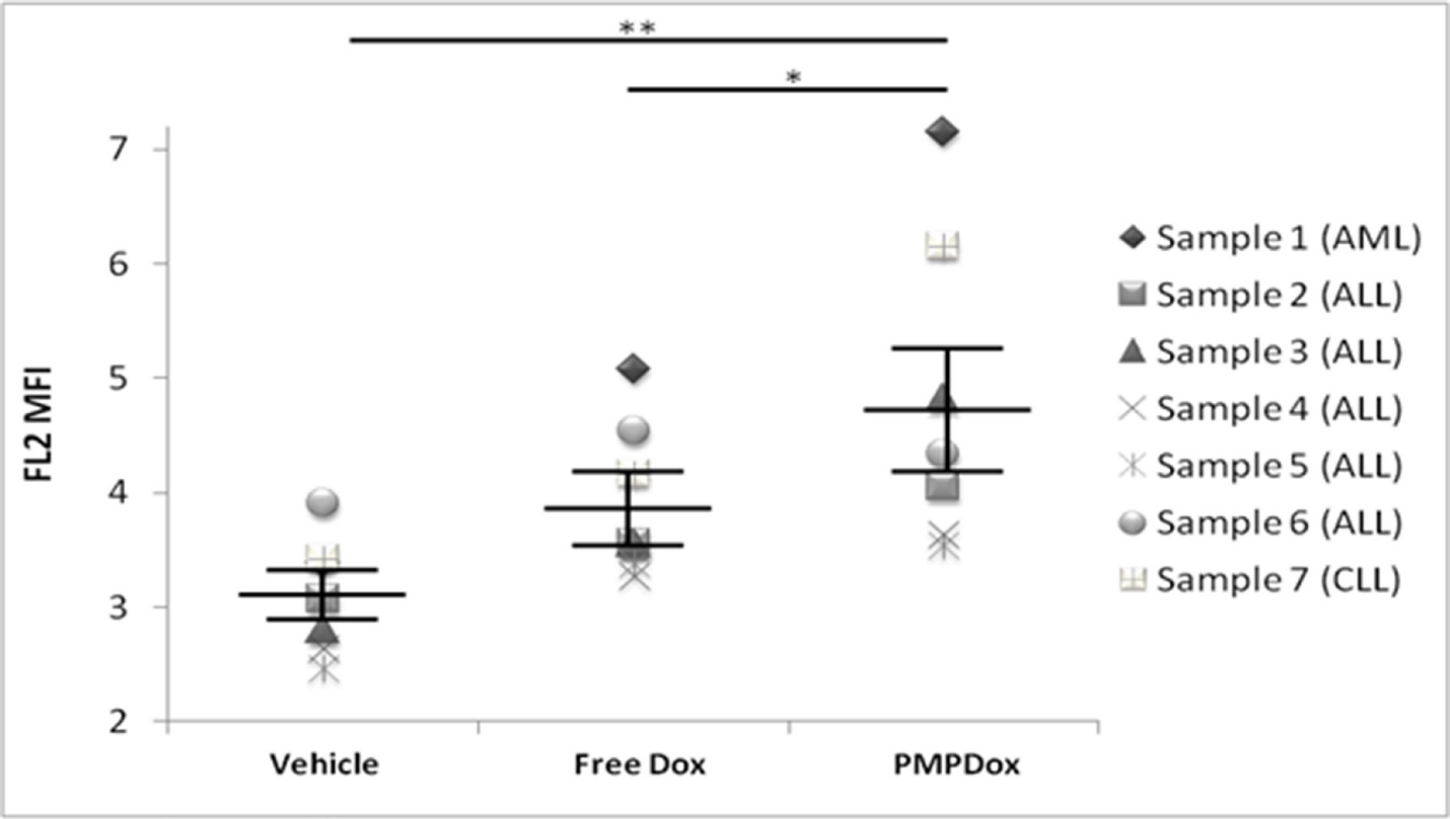
Uptake of Doxorubicin by cancer cells in whole blood samples obtained from patients with leukemia, showing successful and higher uptake of Doxorubicin through PMPDOx compared to free Doxorubicin. Central bars show mean and standard error of mean. (^*^ *P* < 0.05; ^**^ *P* < 0.001; paired *t*-test).

Heparinized whole blood samples obtained from patients with leukemia were incubated with vehicle (buffer without Dox), PMPDox or equivalent amount of free dox. PMPDox successfully delivered drug to leukemia cells (10000 gated cells per sample) in all samples (*p* = 0008, against vehicle) and Dox uptake was higher when carried through PMPDox than the free drug (*p* = 0.042) in six out of seven cases (Figure 8). Such difference in drug delivery was not apparent when normal leukocytes obtained from healthy donors were separately exposed to free drug and PMPDox (Supplementary Figure 6). Furthermore, we did not observe any coagulation of blood or platelet aggregation when PMPs were added to whole blood. The results further supported that, PMP-mediated drug delivery is fairly compatible with whole blood and is the better option for anticancer drug delivery than drug in free form.

## DISCUSSION

We have reported here a quick, easy and high yeild process of engineering human platelets to generate microparticles loaded with doxorubicin (and other compounds), which target leukemia cells and minimize off-target drug delivery. Platelets readily uptake various molecules (which include proteins like fibrinogen and albumin, serotonin and drugs) from surrounding media [26–28], which are repacked into released microparticles [18]. Few studies have earlier reported preparation of drug-loaded extracellular vesicles and exosomes for anticancer therapy but the methods were often long and tedious, requiring complicated cell harvesting, culture and particle isolation steps [13, 29, 30]. On the contrary, procedure described by us for preparation of PMPDox is simple and fast (4 to 5 h) that requires very few reagents. NTA, flow cytometry and fluorimetric charecterization of PMPDox confirmed successful and stable drug loading in PMPs. This method can be widely employed for PMPDox preparation from donated blood or plateletpheresis samples, yeilding large quantitiy drug-loaded PMPs in short period. PMPDox could effectively be stored at −80° C that retains Dox stably upto one month (Supplementary Figure 1).

Leukemia is cancer of leukocytes where premature dysfunctional cells are present in blood. We used HL 60 cells (human aculte myeloid leukemia cell line) to test PMPDox-mediated drug uptake and toxicity. PMPDox quickly bound to HL 60 cells (within 2 min of co-incubation) and drug was delivered at higher concentration as compared to free Dox (Figure 3) attributable to high affinity of PMPs towards leukemia cells. Our experiments suggest that toxicity of PMPDox on target cells was higher than that of free drug (Figure 6). Lower EC50 (0.38 μg/ml) for PMPDox than free Dox (0.58 μg/ml) was consistent with lesser dosage requirement when drug is delivered through PMPs.

As release of free drug is minimal during PMPDox-based delivery, it is less likely to leak out of vascular compartment, as suggested from Transwell system-based experiments (Figure 7), and accumulate at distal off-target organs like liver, kidneys and heart, which in turn would diminish unwanted side effects. In order to establish it, we employed Transwell plates where two chambers of each well were separated by membrane lined with HUVEC cells mimicking endothelial lining. We observed that escape of Dox into lower well (representing extravascular space) was much lower in case of PMPDox (18%) than free drug (51.5%), even in absence of HUVEC lining mimicking damaged endothelium (Figure 7). Reduction of extravascular drug escape can be attributable to barrier effect of HUVEC lining, as well as to faster uptake of PMPDox by leukemia cells (rendering it unavailable), compared to free Dox (Figure 3B), thus validating PMPDox-based approach as safer, with lesser off-target tissue toxicity. In support of this, drug delivery by microvesicle has been already shown to reduce cardiac and liver toxicity in animal models [13].

We also explored possible mechanisms of interaction between PMPs and leukemia cells. Z-stacking / optical slicing by confocal microscopy indicated that, PMPs are not only bound to HL 60 by cell surface attachment but their contents, too, were delivered inside companion cells. PMPs are naturally targeted towards recipient tissues through specific surface ligands like P-selectin and integrins [11, 20, 31]. This obviates the need for implantation of exogenous targeting molecules on PMPs surface unlike the case with synthetic drug-delivery vehicles like liposomes or nano carriers. To explore if HL 60-PMP interaction is through affinity between P-selectin and PSGL-1, we blocked this interaction by pre-incubation with either anti-P-selectin antibody or hydroxyurea, which is known to disrupt P-selectin-PSGL interaction and inhibit platelet phagocytocis by leukocytes [20]. In both cases there was significant albeit partial (~50%) inhibition of PMP uptake in HL 60. PMPDox also interacted with and exhibited cyototoxicity towards K562 and HeLa cells (Supplementary Figure 4), which do not have significant PSGL-1 expression on surface membrane [25]. These observations underscored presence of multiple interfaces apart from P-selectin-PSGL interaction facilitating PMPs uptake by cancer cells.

Compatibility with whole blood is a critical determinant of particle-based drug delivery. Blood components like cells and plasma proteins can seriously impact desired targeting and pharmacokinetics of particles carrying payload of drugs. We evaluated PMPDox-mediated drug delivery in whole blood samples obtained from multiple leukemia patients. PMPDox successfully delivered doxorubicin into leukemia cells when added in whole blood thus validating compatibility of this therapeutic approach. Moreover, PMPDox delivered higher amount of drug to leukemia cells compared with free Dox (of equivalent concentration) in six out of seven samples (Figure 8), which established PMPDox as an efficient and superior anti-leukemia drug delivery vehicle with minimum off-target toxicity. When we compared drug delivery through PMPDox and free Dox (Supplementary Figure 6), it was observed that normal leukocytes also uptake free Dox and PMPDox, but both methods result in similar dose of drug uptake, not higher through PMPDox as observed in leukemia cells. This can be explained by role of extracellular vesicles in cell-cell communication and immunomodulation in cancer [32–34] which is favouring higher drug delivery in leukemia cells via PMPDox.

## MATERIALS AND METHODS

Corning HTS Transwell plate system (# CLS3380-1EA) (96 well, with 1 μm pore size polystyrene membrane support) and dimethyl sulfoxide (DMSO) were procured from Sigma (USA). RPMI 1640 medium, penicillin-streptomycin antibiotic (5,000 U Penicillin and 5 mg/ml Streptomycin in 0.9% normal saline), heat-inactivated fetal bovine serum (FBS), EDTA and PBS (phosphate-buffered saline) were purchased from HiMedia (India). Trypsin-EDTA 1× (0.25%) was from Gibco (Ireland). Doxorubicin (Dox) and 3-(4,5-dimethylthiazol-2-yl)-2,5-diphenyltetrazolium bromide (MTT) were the products of Dabur Pharmaceuticals (India) and SRL Chemicals (India), respectively. Anti P-selectin antibody was purchased from BioLegend (USA) and Calcein-AM was purchased from life technologies (USA). Hydroxyurea was the product of Aimcad Biotech (India). All other reagents used were of analytical grade. Type I deionized water (18.2 MΩ. cm, Millipore, USA) has been used throughout the experiments. Flow cyometry was carried out with BD FACSCalibur employing CellQuest Pro software (BD Biosciences, USA). Fluorimetry was performed with multimodal microplate reader (BioTek model Synergy H1, USA). Nanoparticle Tracking Analysis (NTA) was performed on NanoSight LM10 (Malvern, UK).

### Preparation of washed platelets (WP)

WP samples were prepared from human blood by differential centrifugation as reported earlier [35]. Briefly, 10 ml peripheral venous blood was collected into sterile disposable syringe from multiple healthy donors (as required in respective experiments) under informed consent. Blood was centrifuged at 200 g for 10 min at 22° C to obtain platelet-rich plasma (PRP). PRP was incubated with 1 mM acetylsalicylic acid for 15 min at 37° C. Platelets were sedimented by centrifugation at 600 g for 10 min after addition of 5 mM EDTA. Cells were re-suspended in platelet washing buffer (0.2 M HEPES, 1.38 M NaCl, 29 mM KCl, 10 mM MgCl_2_, 3.6 mM Na_2_HPO_4_, and 10 mM EGTA, pH 6.2, supplemented with 5 mM glucose). Platelets were again centrifuged at 600g for 10 min and finally resuspended in platelet resuspension buffer (0.2 M HEPES, 1.38 M NaCl, 29 mM KCl, 10 mM MgCl_2_, and 3.6 mM Na_2_HPO_4_, pH 7.4, supplemented with 5 mM glucose). Final platelet count was adjusted to 2 × 10^8^ per ml.

### Preparation of Dox-loaded PMPs (PMPDox)

**Figure 9:**
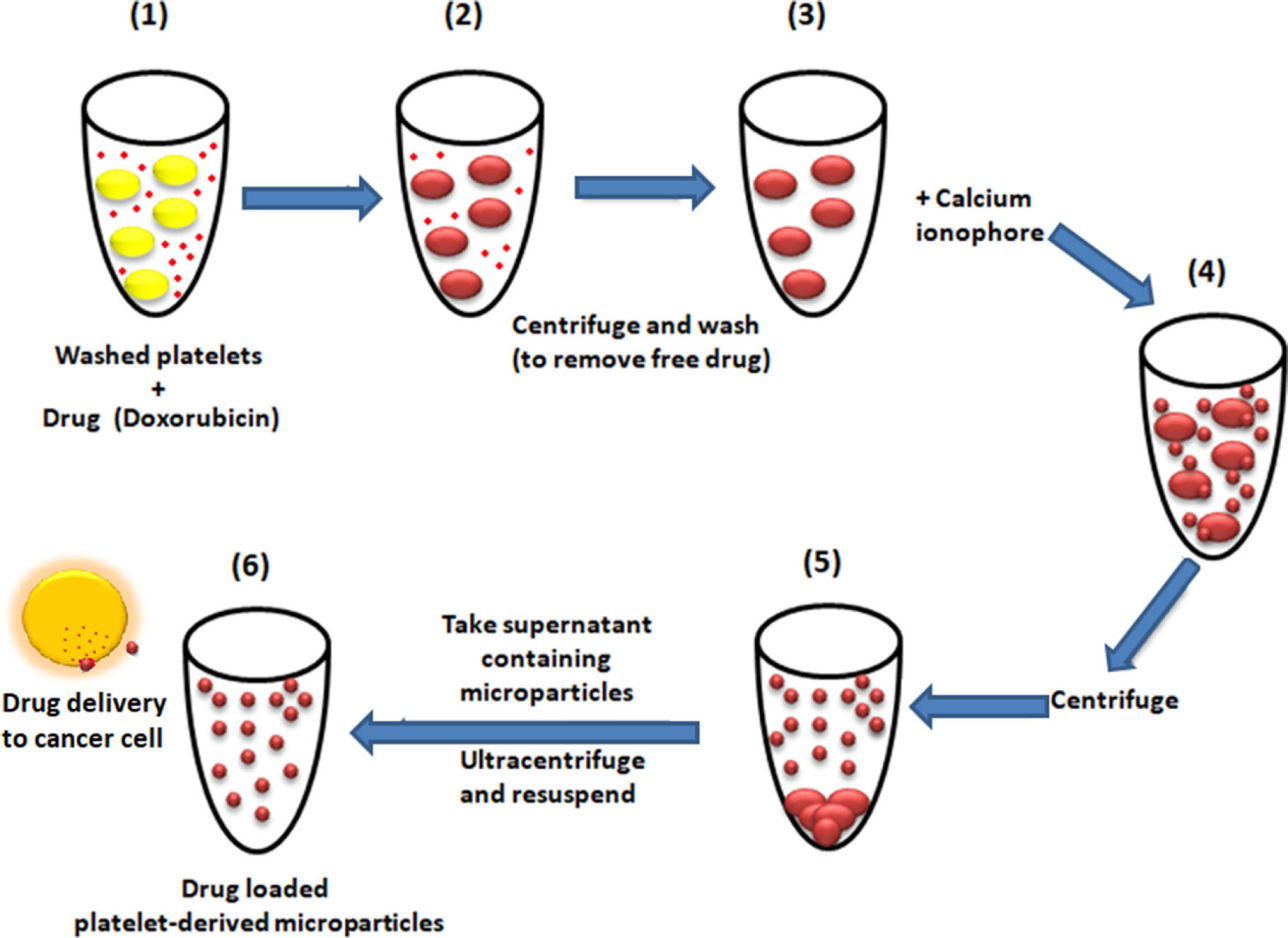
Scheme representing preparation of drug-loaded platelet-derived microparticles, interaction with cancer cell and drug delivery.

We chose to load PMPs with doxorubicin, which is brightly fluorescent compound endowed with effective anticancer properties. Platelets were incubated with 200 μg/ml Dox for 30 min at room temperature [12], washed twice to remove free Dox and finally resuspended in platelet resuspension buffer with 2 mM CaCl_2_. PMP generation was induced by incubation of platelets with 1 μM A23187 for 30 min at 37° C [35]. Cells were pelleted by centrifugation at 1400g for 10 min, followed by 12000 g for 1 min for sedimentation of smaller fragments. Supernatant was centrifuged at 20000g for 30 min to sediment PMPs [18], which were resuspended either in 1 ml platelet resuspension buffer or culture media as required (Figure 9). DOX-loaded PMPs were characterized by NTA, fluorimetry and flow cytometry. Protein content in PMPDox preparations was quantified by Bradford assay. In order to generate Calcein-stained PMP (PMPCalcein), platelets were pre-treated with Calcein-AM (5 μg/ml) in place of Dox and PMPs were generated as discussed above.

### Cell culture

Human leukemia cell lines (HL 60 and K562) were obtained from National Center for Cell Science, Pune, and maintained in RPMI containing 10% FBS and 1× antibiotics (penicillin-100 units per ml and streptomycin 0.1 mg per ml final concentration). Human umbilical vein endothelial cells (HUVECs) were harvested from fresh umbilical cords collected from the Department of Obstetrics of hospital under informed consent. Cords were immediately transferred to PBS under laminar flow, both ends were cut clean and fastened with tubings. Umbilical vein lumen was profusely flushed with PBS, followed by exposure to trypsin-EDTA (0.25%) for 3–5 min. After gentle tapping, contents were collected, diluted with culture media and quickly centrifuged for 5 min at 600 g. Pellet was resuspended in culture media and incubated at 37° C in a 95% humidified air–5% CO_2_ incubator. After 2–3 h, media (along with non-adherent cells) was replaced with fresh media. HUVECs were harvested by Trypsin-EDTA digestion and subjected to Transwell plate experiments.

### Flow cytometry

Samples were diluted with sheath fluid and analyzed with BD FACSCalibur flow cytometer. Appropriate gates were drawn to select microparticles, platelets and cancer cell populations in forward and side scatter plots. Log and linear scales, respectively, were employed for gatings of microparticles and cancer cells. Forward and side scatter voltages were set at E00 and 350, respectively. Fluorescence data were collected by four-quadrant logarithmic amplification using CellQuest Pro software. Fluorescence positive events were collected by running each sample under 488 nm argon-ion laser excitation till fixed event counts were acquired (10000 counts for PMPs as well as cancer cells obtained from clinical samples; 1000-5000 for cultured leukemia cells). Dox loading in microparticles and uptake by cancer cells were analyzed using FL2 channel (bandpass filter, 575/26) in flow cytometer as Dox was found to elicit highest signal with this emission.

### Confocal microscopy

PMPCalcein or PMPDox were incubated with HL 60 cells for 30 min. PMP uptake was studied by confocal microscopy and optical slicing / Z-stacking under Zeiss LSM 700 laser scanning confocal microscope with 1 airy unit pinhole size. Images were acquired and analyzed using ZEN imaging software (Carl Zeiss).

### MTT assay

Cancer cells were plated at 1 × 10^6^ cells per ml (100 ml per well) in 96-well plates and incubated either with vehicle (media without Dox) or drug (either PMPDox or free Dox) for 48 h. Cell viability was measured by addition of 0.5 mg/ml 3-(4,5-dimethylthiazol- 2-yl)-2,5-diphenyltetrazolium bromide (MTT) reagent to each well. Following incubation for 3 h inside CO_2_ incubator, DMSO was added to each well and mixed thoroughly at RT to solubilize formazan crystals. Absorbance at 570 nm was measured using a microplate reader (Biotek model Synergy H1). Viability of vehicle-treated control groups was defined as 100%.

### Transwell plate experiment

HUVECs were grown on porous membrane of Corning HTS Transwell plates till 80-90% confluence, which mimicked endothelial lining separating intravascular (upper chamber) and extravascular (lower chamber) compartments. HL 60 leukemia cells (100 μl from 1 × 10^6^/ml) were added to each chamber. Either PMPDox (0.6 μg Dox/ml) or free Dox (0.6 μg/ml) or equal volume of culture media (control) were added to upper chambers, and plates were incubated for 12 h at 37° C in 95% humidified air–5% CO2 incubator. Dox uptake by HL 60 cells in lower chambers was evaluated by flow cytometry as a measure of drug escape into extravascular tissue (off-target uptake).

### Drug delivery to leukemia cells in clinical samples

Venous blood (0.5 ml each) was collected in heparin-containing vials from seven leukemia patients [acute myeloid leukemia (AML)- one; chronic lymphoblastic leukemia (CLL) one; acute lymphoblastic leukemia (ALL) five] under informed consent. Leukemia diagnosis was confirmed by presence of >90% blast cells with high nuclear-cytoplasmic ratio in peripheral blood and bone marrow examinations. Aliquots (100 μl) of whole blood from each sample were treated either with vehicle (buffer without Dox), or free drug (0.15–0.2 μg/ml DoX), or equivalent PMPDox for 1 h. WBCs were separated using RBC lysis reagent (Puregene), fixed with 2% paraformaldehyde and Dox uptake was evaluated by flow cytometry. Comparison with normal leukocytes was carried out with blood obtained from healthy donors following similar procedure.

All data are representative of at least three independent experiments. Two-tailed Student’s *t*-test was used for evaluation of significance and values of *p* < 0.05 were considered significant. This study was approved by ethical committee of Institute of Medical Sciences, Banaras Hindu University.

## CONCLUSIONS

In this report we discussed a quick and facile top-down approach of generating drug-loaded PMPs that behave as natural vectors targeted against leukemia and other cancers. PMPDox engineered from autologous human platelets are physiological, biocompatible, non-immunogenic and inherently targeted towards cancer cells, thus obviating the need for implantation of exogenous targeting molecules on the particle surface. PMPDox is also capable of carrying multiple therapeutic payloads. Platelets can be easily accessed from blood and harvested in large quantity. PMPDox can be generated in huge number from donor platelets, stored for several days without loss of stability and exhibit targeted delivery and higher toxicity against leukemia cells than free drug. Successful drug delivery and compatibility in whole blood samples from leukemia patients confirmed potential practical application of this approach. Multimodality is also feasible in our approach as multiple anticancer drugs and/or contrasting agent can be loaded into PMP. Reduced extravascular escape of drug from PMPDox would minimize off-target adverse effects including cardiac and hepato- toxicity, making it far safer approach for anticancer therapy.

## SUPPLEMENTARY MATERIALS



## Abbreviations

ALA: δ-aminolevulinic acid
DMSO: dimethyl sulfoxide
Dox: Doxorubicin
EDTA: Ethylenediaminetetraacetic acid
FBS: fetal bovine serum
HUVECs: Human umbilical vein endothelial cells
NTA: Nanoparticle tracking analysis
PBS: phosphate-buffered saline
PMPs: Platelet-Derived Microparticles
PMPDox: Doxorubicin loaded platelet-derived microparticles
PRP: Platelet Rich Plasma
MTT: (4,5-dimethylthiazol-2-yl)-2,5-diphenyltetrazolium bromide
WP: Washed platelets
