# Supplementary material for: Engineered human platelet-derived microparticles as natural vectors for targeted drug delivery
Source: Oncotarget. 2019 Oct 8;10(56):5835–46. doi: 10.18632/oncotarget.27223 (PMC6791386; doi:10.18632/oncotarget.27223)
Supplement: Supplementary file 1 [file oncotarget-10-5835-s001.pdf]

Engineered human platelet-derived microparticles as natural vectors for targeted drug delivery

SUPPLEMENTARY MATERIALS

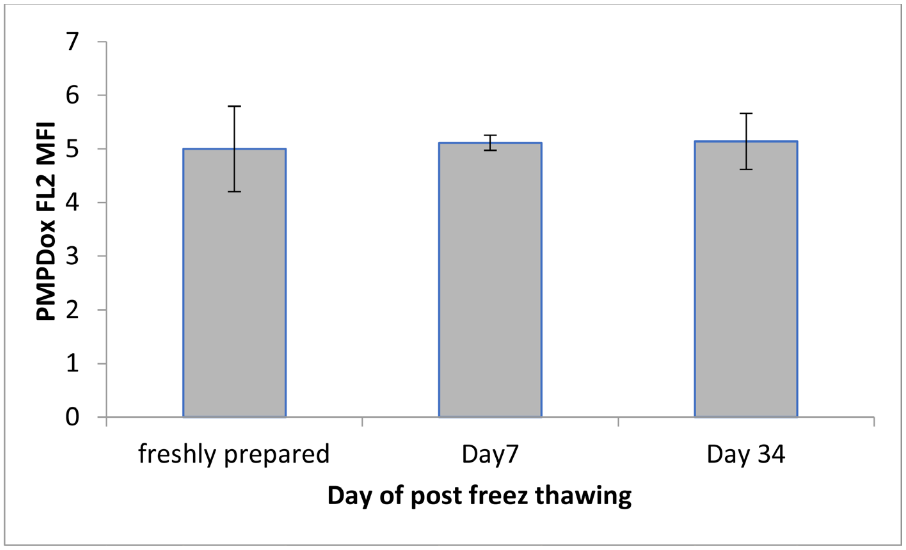

Supplementary Figure 1: Dox fluorescence is well retained in PMPDox samples after storing at −80’C and thawing. Graph shows mean and SD from results of 3 experiments.

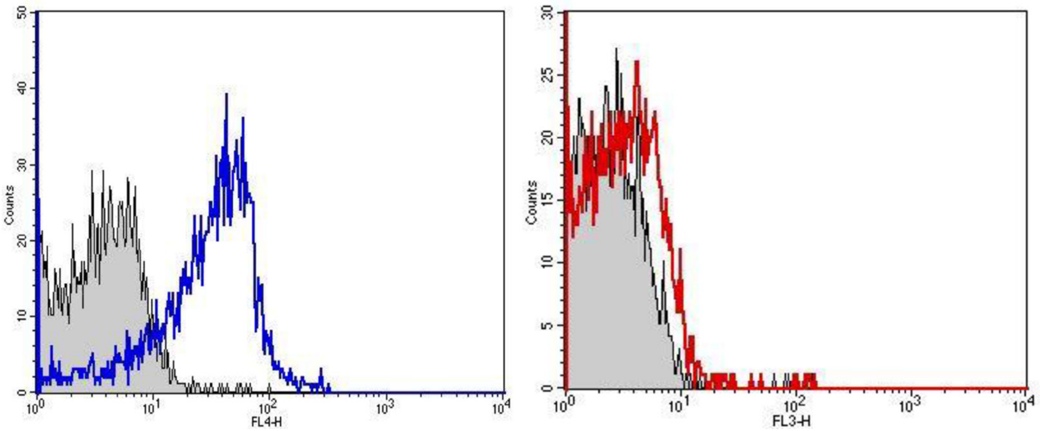

Supplementary Figure 2: Histograms exhibiting successful loading of methylene blue (left panel, blue trace) and ALA (right panel, red trace) in PMPs. Control PMPs (vehicle-treated), shaded trace. Graphs are representative of 3 independent experiments.

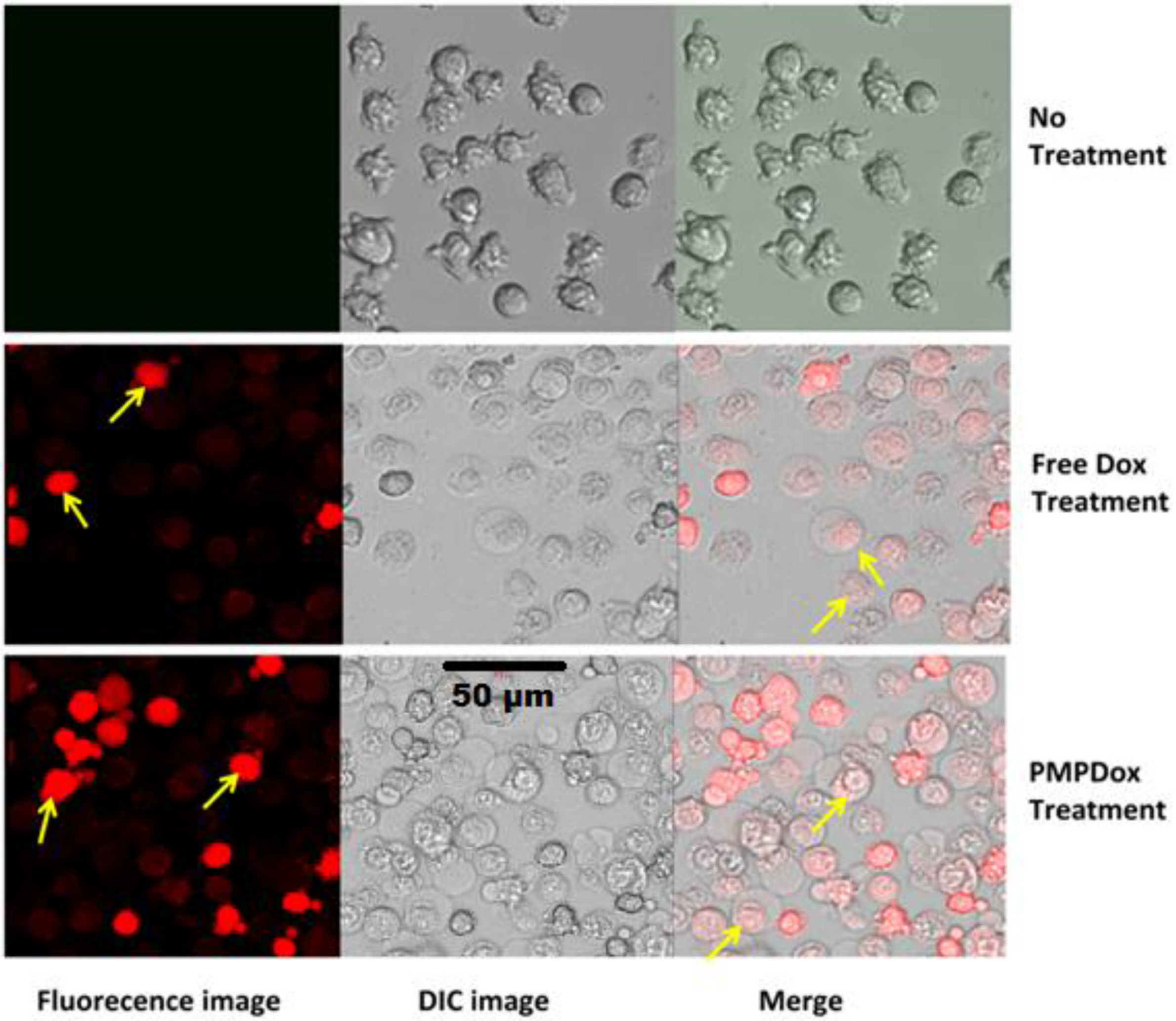

Supplementary Figure 3: Fluorescence microscopy of HL 60 cells without treatment, treatment with free Dox and PMPDox. Nuclei stained with Dox are indicated with arrowheads. Images are representative of 3 independent experiments.

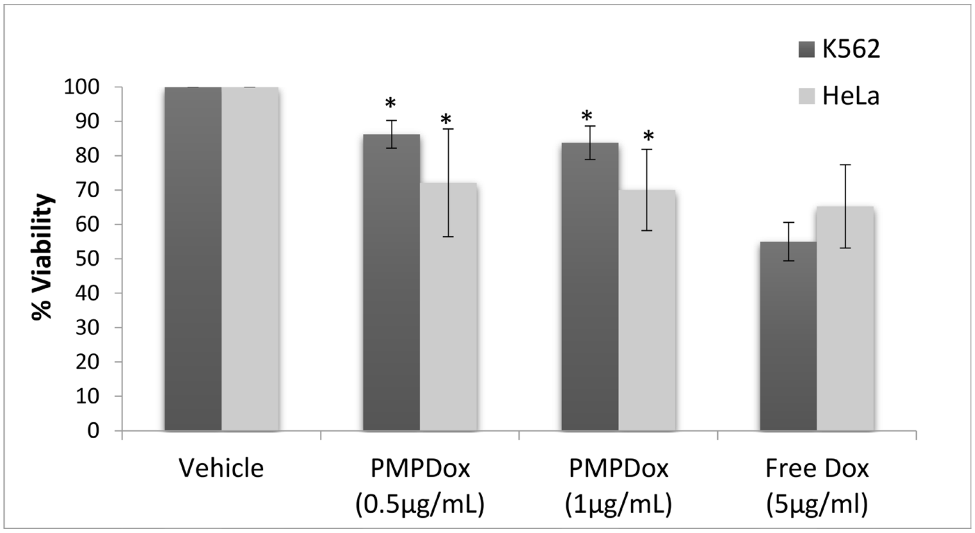

Supplementary Figure 4: PMPDox toxicity in K562 and HeLa cells after 48 h incubation studied by MTT assay. Results represent mean ± SD of 4 sets of experimets. $^{*}P < 0.05$ against vehicle.

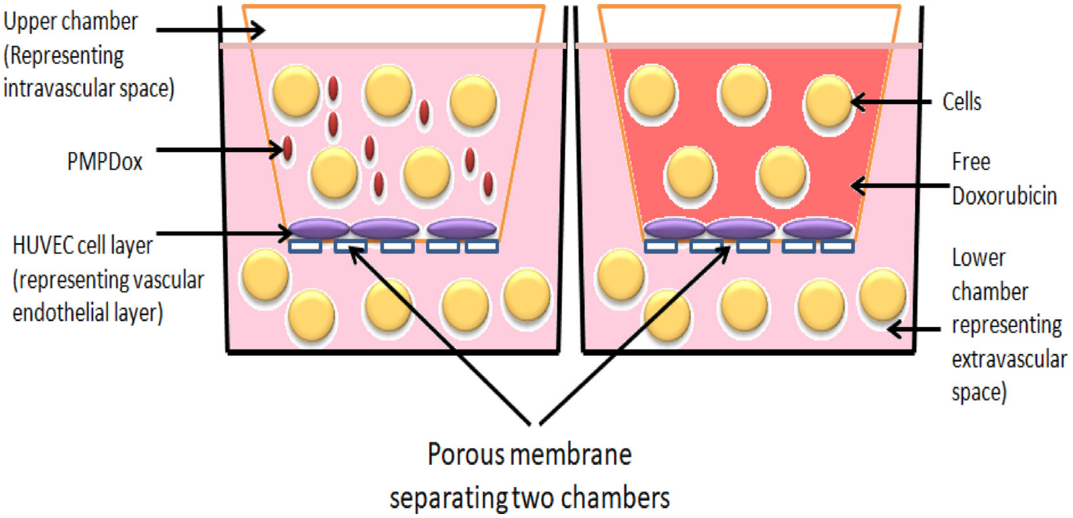

Supplementary Figure 5: Transwell experiment mimicking vascular system. Upper and lower wells were separated by porous membrane lined with HUVEC cells, representing vascular endothelial layer. Upper well represents intravascular while lower well represents extravascular compartments.

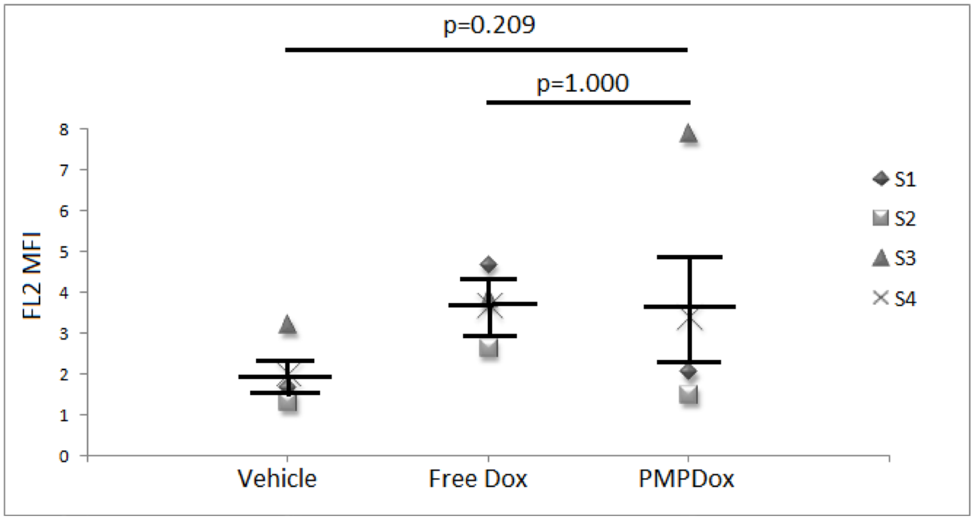

Supplementary Figure 6: Uptake of Dox by normal leukocytes in whole blood samples obtained from 4 healthy donors (S1 to S4), showing uptake of Dox through PMPDOx as well as free Dox. There was no statistically significant difference between both methods (paired *t*-test). Bars show mean and standard error of mean.
